# Robust Low-Complexity WMMSE Precoding Under Imperfect CSI with Per-Antenna Power Constraints

**DOI:** 10.3390/s26010159

**Published:** 2025-12-25

**Authors:** Zijiao Guo, Vaskar Sen, Honggui Deng

**Affiliations:** School of Electronic Information, Central South University, Changsha 410004, China; guozijiao@csu.edu.cn (Z.G.); denghonggui@csu.edu.cn (H.D.)

**Keywords:** massive MU-MIMO, weighted MMSE, per-antenna power constraints, robust precoding, imperfect CSI

## Abstract

Weighted sum-rate (WSR) maximization in downlink massive multi-user multiple-input (MU-MIMO) with per-antenna power constraints (PAPCs) and imperfect channel state information (CSI) is computationally challenging. Classical weighted minimum mean-square error (WMMSE) algorithms, in particular, have per-iteration costs that scale cubically with the number of base-station antennas. This article proposes a robust low-complexity WMMSE-based precoding framework (RLC-WMMSE) tailored for massive MU-MIMO downlink under PAPCs and stochastic CSI mismatch. The algorithm retains the standard WMMSE structure but incorporates three key enhancements: a diagonal dual-regularization scheme that enforces PAPCs via a lightweight projected dual ascent with row-wise safety projection; a Woodbury-based transmit update that replaces the dominant M×M inversion with an (NK)×(NK) symmetric positive-definite solve, greatly reducing the per-iteration complexity; and a hybrid switching mechanism with adaptive damping that blends classical and low-complexity updates to improve robustness and convergence under channel estimation errors. We also analyze computational complexity and signaling overhead for both TDD and FDD deployments. Simulation results over i.i.d. and spatially correlated channels show that the proposed RLC-WMMSE scheme achieves WSR performance close to benchmark WMMSE-PAPCs designs while providing substantial runtime savings and strictly satisfying the per-antenna power limits. These properties make RLC-WMMSE a practical and scalable precoding solution for large-scale MU-MIMO systems in future wireless sensor and communication networks.

## 1. Introduction

Massive MU-MIMO systems are extensively recognized as a fundamental technology for fifth-generation (5G) and subsequent wireless networks. This is due to their ability to significantly spectral efficiency, reliability, and overall network capacity [1,2,3,4,5]. To fully realize these benefits in the downlink, effective precoding is essential to manage multi-user interference. Among linear precoding approaches, the weighted minimum mean-square error (WMMSE) method has gained prominence for achieving near-optimal weighted sum-rate performance in real-world implementations [6,7,8]. Nevertheless, the conventional WMMSE algorithm relies on repeated high-dimensional matrix inversions each iteration, resulting in computational complexity that scales cubically with the number of base station antennas [9,10]. This substantial computational burden challenges its practical deployment in large-scale massive MU-MIMO systems.

In practice, limited pilot resources and severe channel aging in massive MU-MIMO systems, particularly in high-mobility scenarios, make it impractical for the transmitter to obtain accurate channel state information (CSI) for precoding [11], thereby motivating research on robust precoding under imperfect CSI. The imperfect CSI is commonly modeled using a Gaussian-distributed posterior channel model [11,12,13]. Based on this model, robust precoding design can be formulated as an ergodic weighted sum-rate (EWSR) maximization problem subject to per-antenna power constraints (PAPCs). Several iterative robust precoding algorithms have been proposed to address this problem and have demonstrated excellent performance. Furthermore, in many practical BS implementations, each antenna is driven by a separate power amplifier with its own maximum rating [14], so per-antenna power constraints (PAPCs) are often more realistic than a single sum-power constraint (SPC). A simple engineering workaround is to design the precoder under an SPC and then heuristically rescale the columns or rows to satisfy the PAPCs [15], which may cause noticeable WSR loss. To address this, several works have studied WSR maximization directly under PAPCs. For example, zero-forcing (ZF) precoders with per-antenna constraints were analyzed in [16,17,18], but such schemes do not fully exploit the WSR objective because they do not jointly optimize receive filters and user weights. However, many existing approaches rely on fixed approximations or simplified update rules, which can induce non-negligible rate loss and poorer convergence behavior in large-scale regimes [19].

The weighted sum-rate (WSR) maximization problem for the downlink under a sum-power constraint (SPC) is nonconvex and NP-hard [20,21,22]. To address this challenge, the classical weighted minimum mean-square error (WMMSE) algorithm [23] provides an iterative solution by exploiting the fundamental relationship between the mean-square error (MSE) and the signal-to-interference-plus-noise ratio (SINR). This method reformulates WSR maximization as a weighted sum-MSE minimization problem and applies block coordinate descent (BCD) [24], resulting in three closed-form updates for the receive filters, weight matrices, and precoders that converge to a stationary point of the original WSR problem. To alleviate the computational burden, recent research has developed low-complexity variants that approximate WMMSE performance with reduced cost. For example, the rethinking WMMSE (R-WMMSE) algorithm [20] reduces complexity via randomized sketching (data dimensionality reduction). In contrast, our previously proposed LC-WMMSE scheme [25] leverages the structure of the WMMSE update through a Woodbury reformulation combined with a diagonal weight surrogate. While R-WMMSE introduces a probabilistic approximation error due to sketching, LC-WMMSE is deterministic and exhibits per-iteration complexity that scales primarily with the number of data streams rather than the number of BS antennas. Additionally, LC-WMMSE incorporates a hybrid switching mechanism that adaptively blends classical WMMSE updates with lightweight approximations, alongside an adaptive damping strategy that stabilizes the precoder trajectory and accelerates convergence.

Most existing robust and PAPC-aware designs still inherit the high-order matrix inversions of classical WMMSE or rely on generic convex solvers, which become prohibitive for massive MU-MIMO, especially under stochastic CSI mismatch. In contrast, this article proposes a robust low-complexity WMMSE-based framework (RLC-WMMSE) that simultaneously (i) handles imperfect CSI, (ii) enforces PAPCs, and (iii) maintains a low per-iteration complexity. The proposed RLC-WMMSE enforces PAPCs through a lightweight diagonal dual-regularization scheme while keeping the main transmit update in a (NK)×(NK) symmetric positive-definite (SPD) form via a Woodbury identity, so that the dominant cost scales with NK rather than *M*. This makes the method particularly suitable for large-scale MU-MIMO with per-antenna power limits and imperfect CSI. In summary, the main contributions of this paper are as follows:

- We extend the classical WMMSE framework to handle per-antenna power constraints (PAPCs) by introducing a diagonal dual regularization. A diminishing-step projected dual ascent is used to update the per-antenna Lagrange multipliers, and a final row-wise feasibility projection is applied to eliminate any residual PAPC violations. This design keeps all additional variables local to the BS and yields a PAPC-feasible precoder.
- We develop a Woodbury-based reformulation of the transmit update that trades the classical M×M inversion for an (NK)×(NK) symmetric positive-definite (SPD) solve built from a block-diagonal surrogate weight. This reduces the dominant per-iteration complexity in the massive-MIMO regime M≫NK, while retaining a numerically stable Cholesky/LDL implementation.
- We propose a robust low-complexity WMMSE (RLC-WMMSE) update that blends the classical WMMSE precoder with its low-complexity surrogate through an adaptive mixing factor driven by the per-iteration MSE variation. Together with an Armijo-type adaptive damping rule, this hybrid scheme stabilizes the iterations when updates are computed on $\hat{H}$ but performance is evaluated on the true channels H, and enforces sufficient ascent of the monitored WSR in practice.
- Simulation results indicate that the proposed RLC-WMMSE becomes cheaper per iteration than classical WMMSE once M≳cNK for typical system dimensions. In addition, for the PAPC dual loop, we establish a feasibility bound of the form $E\;\left[\max_{m}{\left({\left[PP^{\;H}\right]}_{m,m}-\rho_{m}\right)}_{+}\right]=O\left(\log T/T\right)$, quantifying how the expected PAPC violation decays with the number of outer iterations *T*.
- Through Monte Carlo simulations with both Kronecker-correlated channels and i.i.d. Rayleigh fading, and under various CSI mismatch levels, we show that the proposed RLC-WMMSE achieves near-identical WSR to the WMMSE-PAPCs benchmark while maintaining negligible PAPC violations. At the same time, it exhibits favorable runtime scaling with *M* and clear speedups over classical WMMSE in the large-array regime.

Table 1 summarizes the main design principles of three WMMSE-based precoders discussed in this work. The R-WMMSE approach [20] reduces cost via randomized sketching, solving compressed normal equations. Conversely, the LC-WMMSE method [25] instead exploits problem structure using a Woodbury reformulation and a diagonal-weight surrogate in the transmit step. Our RLC-WMMSE extends LC-WMMSE by adding diagonal dual regularization to handle PAPCs under imperfect CSI, while maintaining an (NK)×(NK) symmetric positive-definite (SPD) solve for the transmit update. The three methods thus differ in update dimension, dominant cost, constraint handling, and approximation source.

The remainder of this article is summarized as follows. Section 2 introduces the system model and problem formulation. Section 3 presents the proposed robust low-complexity WMMSE (RLC-WMMSE) algorithm, including detailed derivations and a complexity analysis. Simulation results are provided and discussed in Section 4. Finally, Section 5 concludes the paper.

## 2. System Model and Problem Formulation

### 2.1. Downlink System Model

We consider a single-cell downlink MU-MIMO system in which a BS with *M* transmit antennas serves *K* users, each equipped with *N* receive antennas. The downlink channel to user *k* is denoted by $H_{k}\in C^{M\times N}$, k=1,…,K, and we model its entries as i.i.d. circularly symmetric complex Gaussian random variables (Rayleigh fading). The transmitted signal is

(1) $$x=\sum_{k=1}^{K}P_{k}s_{k}\in C^{M\times 1},$$

where $P_{k}\in C^{M\times d_{k}}$ is the linear precoder for user *k* and $s_{k}\in C^{d_{k}\times 1}$ is the data symbol vector satisfying $E\left[s_{k}s_{k}^{H}\right]=I_{N}$.

Under flat fading, the received signal at user *k* is

(2) $$y_{k}=H_{k}x+n_{k}=H_{k}\sum_{j=1}^{K}P_{j}s_{j}+n_{k},$$

where $n_{k}∼\mathrm{CN}\left(0,\sigma^{2}I_{N}\right)$ represents additive white Gaussian noise (AWGN). The data vectors $\left\{s_{k}\right\}$ are mutually independent and also independent of the noise vectors $\left\{n_{k}\right\}$.

**Remark** **1** (Scalability in Massive MIMO). *Practical massive MU-MIMO deployments typically satisfy M≫K≥N, meaning the base station is equipped with far more antennas than each user* [26]. *In such configurations, classical algorithms such as WMMSE require large matrix inversions whose computational cost grows rapidly with M. To alleviate this burden, our earlier LC-WMMSE scheme* [25] *integrates hybrid switching and adaptive damping mechanisms, significantly lowering the complexity. These enhancements enable the algorithm to scale efficiently with the BS antenna count, attaining approximately sub-cubic complexity in large-scale regimes.*

### 2.2. Problem Formulation with Imperfect CSI and PAPCs

In downlink MU-MIMO systems, a core design goal is to determine the set of precoders ${\left\{P_{k}\right\}}_{k=1}^{K}$ that maximize the weighted sum-rate (WSR) under a transmit power constraint. Here, $\mu_{k}\geq 0$ represents the weight assigned to user *k*. The WSR is defined as

(3) $$R=\sum_{k=1}^{K}\mu_{k}R_{k},$$

where the achievable rate of user *k* is given by

(4) $$R_{k}=\log_{2}\det\;\left(I_{N}+\Sigma_{k}^{-1}\;H_{k}P_{k}P_{k}^{H}H_{k}^{H}\right),$$

where the interference-plus-noise covariance matrix for user *k* is expressed as

(5) $$\Sigma_{k}=\sigma^{2}I_{N}+\sum_{\begin{array}{c} j=1\\ j\neq k \end{array}}^{K}H_{k}P_{j}P_{j}^{H}H_{k}^{H}.$$

We maximize the WSR over all feasible precoders under per-antenna power constraints (PAPCs), which introduce a different feasibility set and a distinct performance–complexity trade-off compared with the conventional sum-power constraint.

The WSR maximization problem under PAPCs can be expressed as

(6) $$\begin{array}{cc} \max_{\left\{P_{k}\right\}}\; & \sum_{k=1}^{K}\mu_{k}R_{k},\\ s.t.\; & \sum_{k=1}^{K}{\left(P_{k}P_{k}^{H}\right)}_{m,m}\leq \rho_{m}, \end{array}$$

where ${\left[A\right]}_{m,m}$ donates the *m*-th diagonal element of matrix A. The constraints in Equation (6) ensure that the transmit power at the *m*-th antenna of the BS does not exceed $\rho_{m}$. Solving the WSR maximization problem in Equation (6) is challenging due to the objective function being highly nonlinear and nonconvex. Furthermore, as shown in [22], this problem is NP-hard, as summarized in the following proposition.

**Proposition** **1** (NP-hardness of WSR Maximization). *The downlink weighted sum-rate maximization problem under a sum-power constraint is known to be NP-hard *[21,22]*. Since PAPCs are more restrictive than an SPC, the PAPC formulation (6) is also NP-hard.*

We adopt the standard imperfect-CSI model $H_{k}\;=\;{\hat{H}}_{k}\;+\;\Delta_{k}$, $\Delta_{k}∼\mathrm{CN}\;\left(0,\;\varepsilon \;I_{M\times N}\right)$, k=1,…,K, where ${\hat{H}}_{k}$ is the channel estimate and ε sets the estimation NMSE; we use $E\;\left[∥\Delta_{k}{∥}_{F}^{2}\right]/E\;\left[∥H_{k}{∥}_{F}^{2}\right]=\varepsilon$. Given nonnegative user weights $\left\{\mu_{k}\right\}$, the weighted sum-rate (WSR) for a channel realization is $R\left(\left\{P_{k}\right\};H\right)=\sum_{k=1}^{K}\mu_{k}R_{k}\left(\left\{P_{j}\right\};H\right)$, with $R_{k}$ and $\Sigma_{k}$ defined in Equations (4) and (5).

Imperfect CSI model: The additive Gaussian CSIT error model above is widely used in robust precoding/beamforming because it is tractable and captures the aggregate impact of estimation noise, quantization, and residual uncertainty; see, e.g., [11,12,13].

**Remark** **2** (CSI outdatedness). *In practice, CSIT can also be outdated due to feedback/processing delay, which is often modeled by a first-order Gauss–Markov evolution $H_{k}\left[n\right]=\alpha H_{k}\left[n-1\right]+\sqrt{1-\alpha^{2}}W_{k}\left[n\right]$* [2,27]*. In this work, we focus on the additive error model for algorithm design and analysis, and in simulations, we sweep ϵ to represent different CSI qualities.*

Under per-antenna power constraints (PAPCs), the feasible precoder set is

(7) $$P_{\mathrm{PAPC}}≜\left\{{\left\{P_{k}\right\}}_{k=1}^{K}:\;{\left[\sum_{k=1}^{K}P_{k}P_{k}^{\;H}\right]}_{m,m}\leq \rho_{m},\;\;m=1,\ldots ,M\right\},$$

where $\rho ={\left[\rho_{1},\ldots ,\rho_{M}\right]}^{\;⊤}$ collects the per-antenna budgets. For apples-to-apples comparisons with the SPC case, we set $\sum_{m=1}^{M}\rho_{m}=P_{\max}$.

A practical “plug-in” (mismatch) design maximizes the WSR built on the channel estimates ${\hat{H}}_{k}$ while enforcing PAPCs:

(8) $$\begin{array}{cc} \underset{\left\{P_{k}\right\}\in P_{\mathrm{PAPC}}}{\mathrm{maximize}}\;\; & \tilde{R}\left(\left\{P_{k}\right\};\hat{H}\right)\;≜\;\sum_{k=1}^{K}\mu_{k}\log_{2}\det\;\left(I_{N}+{\tilde{\Sigma }}_{k}^{-1}\;{\hat{H}}_{k}P_{k}P_{k}^{\;H}{\hat{H}}_{k}^{\;H}\right)\\ \mathrm{where}\;\; & {\tilde{\Sigma }}_{k}\;=\;\sigma^{2}I_{N}\;+\;\;\sum_{j\neq k}{\hat{H}}_{k}P_{j}P_{j}^{\;H}{\hat{H}}_{k}^{\;H}, \end{array}$$

with $I_{N}$ the N×N identity. Problem Equation (8) is nonconvex and NP-hard. We solve it via alternating WMMSE updates on $\hat{H}$ combined with a dual-based enforcement of Equation (7) and a Woodbury low-complexity step; the final performance is reported by evaluating $R(\left\{P_{k}\right\};H)$ on the true channel. All symbols used in this paper are summarized in Table 2.

## 3. Proposed Robust LC-WMMSE Algorithm

### 3.1. The Classical WMMSE Algorithm

The WMMSE framework is widely used for WSR optimization [20]. In this section, we briefly revisit the classical WMMSE approach [23,28] from an optimization perspective, where the MSE serves as an auxiliary variable rather than a physical metric. Since the WSR problem in (6) is nonconvex, we leverage the well-known rate–MSE equivalence to rewrite it as a weighted sum-MSE minimization, which admits an efficient BCD procedure [24]; the problem can be reformulated as

(9) $$\begin{array}{cc} \min_{\left\{W_{k},U_{k},P_{k}\right\}}\; & \;\sum_{k=1}^{K}\mu_{k}\left(\mathrm{Tr}\left(W_{k}E_{k}\right)-\log\det\left(W_{k}\right)\right)\\ s.t.\;\; & \sum_{k=1}^{K}{∥P_{k}∥}_{F}^{2}\leq P_{\max} \end{array}$$

subject to the same transmit power constraint specified in Equation (9). Here, $\mu_{k}\geq 0$ denotes the priority weight associated with user *k*, and the mean-square error (MSE) matrix $E_{k}$ for user *k* is defined as

(10) $$E_{k}\;=\;E\;\left[\;\left(U_{k}^{H}y_{k}-s_{k}\right)\;{\left(U_{k}^{H}y_{k}-s_{k}\right)}^{H}\right],$$

where $E_{k}$ denotes the user-*k* mean-square error (MSE) matrix. Here, $U_{k}\in C^{N\times d_{k}}$ is the linear receive filter and $W_{k}\in C^{d_{k}\times d_{k}}$ is the MSE weight matrix. Both variables are updated jointly with the precoders $\left\{P_{k}\right\}$. By expanding $E_{k}$, we obtain

(11) $$\begin{array}{c} E_{k}\;=\;I_{N}-U_{k}^{\;H}{\hat{H}}_{k}P_{k}-{\left(U_{k}^{\;H}{\hat{H}}_{k}P_{k}\right)}^{\;H}+\sum_{j=1}^{K}U_{k}^{\;H}{\hat{H}}_{k}P_{j}P_{j}^{\;H}{\hat{H}}_{k}^{\;H}U_{k}+\sigma^{2}U_{k}^{\;H}U_{k}. \end{array}$$

As shown in [23], the reformulated problem in Equation (9) is nonconvex in the joint variable set $\left\{P_{k},U_{k},W_{k}\right\}$. Nonetheless, it is convex in each variable block with the others fixed, which enables an efficient alternating optimization scheme summarized below:

(12) $$U_{k}\;=\;{\left(\sum_{j=1}^{K}{\hat{H}}_{k}P_{j}P_{j}^{\;H}{\hat{H}}_{k}^{\;H}+\sigma^{2}I_{N}\right)}^{\;-1}{\hat{H}}_{k}P_{k},\;\forall k.$$

Keeping the remaining two variable blocks unchanged, the weight matrix $W_{k}$ admits the following closed-form update:

(13) $$\begin{array}{c} W_{k}=\mu_{k}{\left(E_{k}+\varepsilon \;I_{d_{k}}\right)}^{-1},\;E_{k}\in C^{d_{k}\times d_{k}},\;\forall k. \end{array}$$

For given receive filters $\left\{U_{k}\right\}$ and weight matrices $\left\{W_{k}\right\}$, the precoders $\left\{P_{k}\right\}$ are updated by the solution of a convex quadratic problem:

(14) $$P=\arg\min_{P}\;\frac{1}{2}\langle P,AP\rangle -ℜ\left\{\langle B,P\rangle \right\},$$

with the corresponding normal equation AP=B.

(15) $$\begin{array}{c} A≜\sigma^{2}I_{M}+\sum_{k=1}^{K}{\hat{H}}_{k}U_{k}W_{k}U_{k}^{H}{\hat{H}}_{k}^{H}\in C^{M\times M}, \end{array}$$

(16) $$\begin{array}{c} B≜\left[\;{\hat{H}}_{1}U_{1}W_{1}\;\ldots \;{\hat{H}}_{K}U_{K}W_{K}\;\right]\in C^{M\times D},\;D=\sum_{k=1}^{K}d_{k}. \end{array}$$

Here, $A\in C^{M\times M}$, $B\in C^{M\times D}$, $P\in C^{M\times D}$, and $P_{k}\in C^{M\times d_{k}}$. Using the definitions of A and B in (15) and (16), the optimality condition AP=B gives the unique solution $P=A^{-1}B$, which can be written per user as

(17) $$\;P=A^{-1}B\;\;⟺\;\;P_{k}=A^{-1}\left({\hat{H}}_{k}U_{k}W_{k}\right),\;k=1,\ldots ,K.$$

While A is shared by all users, the blocks on the right-hand side, ${\hat{H}}_{k}U_{k}W_{k}$, depend on *k*; consequently, the precoder blocks $P_{k}$ are user-specific. Since $\sigma^{2}>0$ and ε>0 in (13), A≻0 (Hermitian), and the associated system admits a unique solution. Now, we replace the SPC update in Equation (17) by a per-antenna constrained step; for a dual vector λ⪰0, define

(18) $$P\left(\lambda \right)={\left(A+\mathrm{diag}(\lambda )\right)}^{-1}B,$$

and choose λ so that each antenna power satisfies

(19) $${\left[P\left(\lambda \right)P{\left(\lambda \right)}^{\;H}\right]}_{m,m}\leq \rho_{m},\;m=1,\ldots ,M.$$

A projected dual update is

(20) $$\lambda^{\left(t+1\right)}={\left[\lambda^{\left(t\right)}+\eta_{t}\left(\mathrm{diag}(PP^{\;H})-\rho \right)\right]}_{+},\;\eta_{t}=\eta_{0}/\sqrt{t}.$$

If a tiny residual remains, apply the row-wise safety projection

(21) $$P\leftarrow S\;P,\;S=\mathrm{diag}\;{\left(\min\;\left\{1,\sqrt{\frac{\rho_{m}}{{\left[PP^{\;H}\right]}_{m,m}}}\right\}\right)}_{m=1}^{M}.$$

**Remark** **3.** *If $\sum_{m=1}^{M}\rho_{m}=P_{\max}$ and λ=λ1, Equation (18) reduces to the SPC form in Equation (17).*

The classical WMMSE algorithm requires solving an M×M linear system in every outer iteration, which entails cubic work $O(M^{3})$ and becomes the dominant bottleneck when *M* is large. With per-antenna constraints the transmit update must also be regularized by a diagonal dual term, but the cost is still governed by the M×M solve. This motivates a low-complexity variant that avoids the big inversion. In the next subsection, we construct a Woodbury update that shifts the solve to an (NK)×(NK) system and enforces PAPCs with a light projected dual loop, yielding a favorable crossover when M≳cNK while preserving the WSR performance under CSI mismatch.

### 3.2. Proposed RLC-WMMSE

In this subsection, we develop an efficient iterative solution to the PAPC-constrained WSR maximization problem in Equation (8) using the WMMSE framework. The proposed robust low-complexity WMMSE (RLC-WMMSE) alternates standard WMMSE updates (on $\hat{H}$) with a light dual update to enforce PAPCs and a Woodbury-based transmit step; hybrid switching and adaptive damping are employed for stability under CSI mismatch.

#### The RLC-WMMSE Algorithm Problem Reformulation

In the proposed robust LC-WMMSE, the M×M matrix inversion in (15)–(17) is avoided by applying the Woodbury identity [29], which reduces the update to solving an (NK)×(NK) system. As a result, the dominant per-iteration complexity decreases from $O(M^{3})$ to $O\;\left(M{\left(NK\right)}^{2}+{\left(NK\right)}^{3}\right)$ in the massive-MIMO setting M≫NK.

- Update the hybrid transmit precoder: At iteration *t*, we construct $P^{\left(t\right)}$ by convexly combining the classical WMMSE precoder $P_{\mathrm{WMMSE}}^{\left(t\right)}$ and the LC precoder $P_{\mathrm{LC}}^{\left(t\right)}$ as follows:
  (22) $$P^{\left(t\right)}\;=\;\omega^{\left(t\right)}\;P_{\mathrm{WMMSE}}^{\left(t\right)}\;+\;\left(1-\omega^{\left(t\right)}\right)\;P_{\mathrm{LC}}^{\left(t\right)},$$
  where $\omega^{\left(t\right)}\in \left[0,1\right]$ is an adaptive switch:
  (23) $$\omega^{\left(t\right)}\;=\;\frac{{∥\Delta E^{\left(t\right)}∥}_{F}}{{∥\Delta E^{\left(t\right)}∥}_{F}+\kappa },\;\Delta E^{\left(t\right)}\;=\;\sum_{k=1}^{K}\;\left(E_{k}^{\left(t\right)}-E_{k}^{\left(t-1\right)}\right),$$
  with a small smoothing constant κ>0 (we use $\kappa =10^{-3}$ unless stated otherwise). When $∥\Delta E^{\left(t\right)}{∥}_{F}$ is large (far from a fixed point), $\omega^{\left(t\right)}\;\approx 1$ favors $P_{\mathrm{WMMSE}}^{\left(t\right)}$; near convergence, it gradually shifts to the LC update to save time.
  Diagonal weight and Woodbury build.
  We approximate the full WMMSE weight by its diagonal,
  (24) $$D_{k}^{\left(t\right)}\;=\;\mathrm{diag}\;\left(\mathrm{diag}(W_{k}^{\left(t\right)})\right)≻0,\;D_{k}^{\left(t\right)}\in C^{d_{k}\times d_{k}},\;k=1,\ldots ,K,$$
  which preserves positive-definiteness while removing inter-stream couplings. Using $U_{k}^{\left(t\right)}$ and $D_{k}^{\left(t\right)}$, we form the block diagonal matrix
  (25) $$S^{\left(t\right)}\;=\;\mathrm{blkdiag}\;\left(U_{1}^{\left(t\right)}D_{1}^{\left(t\right)}U_{1}^{{\left(t\right)}^{H}},\ldots ,U_{K}^{\left(t\right)}D_{K}^{\left(t\right)}U_{K}^{{\left(t\right)}^{H}}\right)\in C^{\left(NK)\times (NK\right)},\;S^{\left(t\right)}≻0,$$
  and stack the channels horizontally,
  (26) $$\hat{H}\;=\;\left[\;{\hat{H}}_{1},\ldots ,{\hat{H}}_{K}\;\right]\in C^{M\times (NK)}.$$
  PAPC enforcement via diagonal dual regularization.
  Under per-antenna power constraints, we replace the global Frobenius normalization by a diagonal dual regularization. At iteration *t*, construct $A^{\left(t\right)}$ and $B^{\left(t\right)}$ from the current $\left(U_{k}^{\left(t\right)},D_{k}^{\left(t\right)},w_{k}^{\left(t\right)}\right)$ as in (27) and (28). Let
  (27) $$\begin{array}{c} A^{\left(t\right)}=\sigma^{2}I_{M}+\sum_{k=1}^{K}{\hat{H}}_{k}\;U_{k}^{\left(t\right)}\;W_{k}^{\left(t\right)}\;U_{k}^{{\left(t\right)}^{H}}\;{\hat{H}}_{k}^{H}, \end{array}$$
  (28) $$B^{\left(t\right)}=\;\left[\;{\hat{H}}_{1}U_{1}^{\left(t\right)}D_{1}^{\left(t\right)},\ldots ,{\hat{H}}_{K}U_{K}^{\left(t\right)}D_{K}^{\left(t\right)}\;\right]\in C^{M\times D}.$$
  With these, the LC step inverts an (NK)×(NK) SPD matrix (via Cholesky) instead of an M×M system, yielding the usual Woodbury speedup when M≫NK. For a dual vector λ⪰0, define
  (29) $$P^{\left(t\right)}\left(\lambda \right)\;=\;{\left(A^{\left(t\right)}+\mathrm{diag}\left(\lambda \right)\right)}^{-1}B^{\left(t\right)},$$
  and choose λ so that each antenna satisfies the PAPC ${\left[P^{\left(t\right)}\;P^{{\left(t\right)}^{H}}\right]}_{m,m}\leq \rho_{m}$, m=1,…,M. A lightweight projected dual ascent that works well in practice is
  (30) $$\lambda^{\left(i+1\right)}\;=\;{\left[\lambda^{\left(i\right)}+\eta_{i}\left(\mathrm{diag}(P^{\left(t\right)}P^{{\left(t\right)}^{H}})-\rho \right)\right]}_{+},\;\eta_{i}=\frac{\eta_{0}}{\sqrt{i}},$$
  with ${\left[\cdot \right]}_{+}$ denoting projection onto $R_{\geq 0}^{M}$ and $\rho ={\left[\rho_{1},\ldots ,\rho_{M}\right]}^{T}$. If a tiny residual remains after the inner loop, apply the row-wise safety projection
  (31) $$P^{\left(t\right)}\;\leftarrow \;S\;P^{\left(t\right)},\;S=\mathrm{diag}\;{\left(\min\;\left\{1,\sqrt{\frac{\rho_{m}}{{\left[P^{\left(t\right)}P^{{\left(t\right)}^{H}}\right]}_{m,m}}}\right\}\right)}_{m=1}^{M}.$$
  The row scaling in (31) is not an ad-hoc post-processing; it is the exact row-wise Euclidean projection onto the PAPC-feasible set $P≜\{P:\;∥P_{m,:}{∥}_{2}^{2}\leq \rho_{m},\;\forall m\}$, since the constraints decouple across rows. Concretely, for any $\hat{P}$, $\Pi_{P}\left(\hat{P}\right)$ is obtained by $P_{m,:}=\min\;\left(1,\sqrt{\rho_{m}}/{∥{\hat{P}}_{m,:}∥}_{2}\right){\hat{P}}_{m,:}$. Thus, every iterate is feasible (up to numerical tolerance), and Armijo acceptance is evaluated on the projected (feasible) iterate.
  Notes. (i) Equations (29)–(31) enforce PAPCs without any global Frobenius rescaling, so this section supersedes the SPC normalization previously used after Equation (28). (ii) If $\sum_{m}\rho_{m}=P_{\max}$ and λ=λ1, Equation (29) reduces to the SPC form (recovering the classical update).
- Adaptive Damping with Robust PAPCs: To stabilize the outer iterations under CSI mismatch, we adopt the adaptive damping mechanism originally proposed for the LC-WMMSE algorithm in [25] and extend it to the robust PAPC setting. Let $\tilde{R}\left(\left\{P_{k}\right\};\hat{H}\right)$ denote the estimated WSR evaluated on the imperfect CSI $\hat{H}$. At iteration *t*, we measure the change in the estimated WSR as
  (32) $$\Delta R^{\left(t\right)}≜\left|\tilde{R}\left(\left\{P_{k}^{\left(t\right)}\right\};\hat{H}\right)-\tilde{R}\left(\left\{P_{k}^{\left(t-1\right)}\right\};\hat{H}\right)\right|,\;{\bar{\Delta }}^{\left(t\right)}≜\frac{\Delta R^{\left(t\right)}}{\max\{1,\;|\tilde{R}\left(\left\{P_{k}^{\left(t\right)}\right\};\hat{H}\right)|\}}.$$
  We then choose a mixing factor
  (33) $$\alpha^{\left(t\right)}=\mathrm{clip}\;\left(1-\frac{{\bar{\Delta }}^{\left(t\right)}}{\eta_{t}},\;\alpha_{\min},\;\alpha_{\max}\right),\;\eta_{t}=\frac{\eta_{0}}{\sqrt{t}},$$
  where clip(x,a,b)≜min{max{x,a},b} and typical values are $\alpha_{\min}\in \left[0.1,0.3\right]$, $\alpha_{\max}\in \left[0.9,0.98\right]$, and $\eta_{0}\in \left[10^{-2},10^{-1}\right]$. Let ${\hat{P}}^{\left(t\right)}$ denote the undamped precoder returned by the robust LC-WMMSE (RLC) update at iteration *t*. The damped update is
  (34) $$P^{\left(t\right)}\leftarrow \alpha^{\left(t\right)}P^{\left(t-1\right)}+\left(1-\alpha^{\left(t\right)}\right){\hat{P}}^{\left(t\right)}.$$
  Optionally, we apply a short Armijo backtracking (up to a few trials) on $\alpha^{\left(t\right)}$ to enforce monotone ascent of the estimated objective, i.e., $\hat{R}\left(\left\{P_{k}^{\left(t\right)}\right\};\hat{H}\right)\geq \hat{R}\left(\left\{P_{k}^{\left(t-1\right)}\right\};\hat{H}\right)$. This schedule reduces the step size when the iterate is far from stationarity (large ${\bar{\Delta }}^{\left(t\right)}$) and allows larger steps near convergence. Final performance is always reported as $R(\left\{P_{k}^{\left(t\right)}\right\};H)$ on the true channel H for fairness.

### 3.3. Proposed RLC-WMMSE Updates Precoder with PAPCs and Imperfect CSI

The proposed robust low-complexity WMMSE (RLC-WMMSE) precoding algorithm follows the standard three-block WMMSE structure, but computes all updates with the channel estimates ${\hat{H}}_{k}$ and enforces per-antenna power constraints (PAPCs) via the diagonal dual map described in Section 3.2.

- Receive Filter Update $U_{k}^{\left(t\right)}$: At iteration *t*, the receive filter for user *k* is
  (35) $$U_{k}^{\left(t\right)}={\left({\hat{H}}_{k}^{H}\;S_{x}^{\left(t-1\right)}\;{\hat{H}}_{k}+\sigma^{2}I_{N}\right)}^{-1}{\hat{H}}_{k}^{H}\;P_{k}^{\left(t-1\right)},\;\forall k,$$
  where the BS transmit covariance from the previous iterate is $S_{x}^{\left(t-1\right)}=\sum_{j=1}^{K}P_{j}^{\left(t-1\right)}$$P_{j}^{(t-1)H}\in C^{M\times M}$. The matrix inside the inverse is Hermitian positive-definite, so Equation (35) is well posed.
- Weight Matrix Update $W_{k}^{\left(t\right)}$: With the MSE matrix $E_{k}^{\left(t\right)}$ (defined earlier), we set
  (36) $$W_{k}^{\left(t\right)}\;=\;\mu_{k}{\left(E_{k}^{\left(t\right)}+\varepsilon I_{N}\right)}^{-1},\;\varepsilon >0\;\;\forall k,$$
  which yields a positive-definite weight; in the LC branch, we use its diagonal surrogate as shown in Equations (24)–(28); we form $S^{\left(t\right)}$ and stack $\hat{H}$ to obtain the Woodbury update.
- Transmit Precoder Update $P^{\left(t\right)}$: Using the block-diagonal matrix $S^{\left(t\right)}$ and the stacked channel $\hat{H}=\left[{\hat{H}}_{1},\ldots ,{\hat{H}}_{K}\right]$ (both defined earlier), the Woodbury step gives the LC precoder in closed form as
  (37) $$P^{\left(t\right)}=\frac{1}{\sigma^{2}}\left[\;B^{\left(t\right)}-\hat{H}{\left({\left(S^{\left(t\right)}\right)}^{-1}+\frac{1}{\sigma^{2}}{\hat{H}}^{H}\hat{H}\right)}^{-1}\frac{1}{\sigma^{2}}{\hat{H}}^{H}B^{\left(t\right)}\right]\in C^{M\times D},$$
  with $B^{\left(t\right)}=\left[\;{\hat{H}}_{1}U_{1}^{\left(t\right)}D_{1}^{\left(t\right)},\ldots ,{\hat{H}}_{K}U_{K}^{\left(t\right)}D_{K}^{\left(t\right)}\;\right]$ and $S^{\left(t\right)}=\mathrm{blkdiag}\left(U_{k}^{\left(t\right)}D_{k}^{\left(t\right)}U_{k}^{(t)H}\right)$. This moves the inversion from size *M* to size NK, yielding per-iteration cost $O\;\left(M{\left(NK\right)}^{2}\right)+{\left(NK\right)}^{3}$ instead of $O(M^{3})$ when M≫NK.

#### PAPC Enforcement (Replaces SPC Rescaling)

Rather than the global Frobenius normalization, we enforce PAPCs via the diagonal dual regularization described in Section 3.2: the classical and LC right-hand sides are evaluated through $P^{\left(t\right)}\left(\lambda \right)={\left(A^{\left(t\right)}+\mathrm{diag}\left(\lambda \right)\right)}^{-1}B^{\left(t\right)}$ Equation (18), where λ is updated by the projected dual step in Equation (20); if a tiny residual remains, we apply the row-wise safety projection in Equation (21). This entirely replaces the SPC normalization.

### 3.4. Convergence and Feasibility Analysis

The classical WMMSE alternates minimization of a convex quadratic surrogate and achieves monotone ascent of the WSR [23]. In our robust low-complexity variant, at iteration *t*, we form the transmit quadratic $Q^{\left(t\right)}\left(P\right)=\frac{1}{2}\langle P,A^{\left(t\right)}P\rangle -ℜ\left\{\langle B^{\left(t\right)},P\rangle \right\}$, from the pairs ${\left\{\left(U_{k}^{\left(t\right)},W_{k}^{\left(t\right)}\right)\right\}}_{k=1}^{K}$ computed on the estimates $\hat{H}$, and obtain a candidate update ${\hat{P}}^{\left(t\right)}\in \left\{P_{\mathrm{WMMSE}}^{\left(t\right)},\;P_{\mathrm{LC}}^{\left(t\right)}\right\}$, the LC candidate uses the diagonal surrogate with $D_{k}^{\left(t\right)}\;=\;\mathrm{diag}\left(W_{k}^{\left(t\right)}\right)$. We then apply the damped step

(38) $$P^{\left(t+1\right)}\;=\;\left(1-\alpha^{\left(t\right)}\right)\;P^{\left(t\right)}\;+\;\alpha^{\left(t\right)}\;{\hat{P}}^{\left(t\right)},\;\alpha^{\left(t\right)}\in \left(0,1\right],$$

where $\alpha^{\left(t\right)}$ is chosen by Armijo backtracking on the true-channel objective WSR(P;H) evaluated at the feasible iterate $P^{\left(t+1\right)}$.

**Proposition** **2** (Monotone WSR and limit-point stationarity). *With Armijo acceptance evaluated on the feasible iterate $P^{\left(t+1\right)}$, the objective sequence $\left\{\mathrm{WSR}\left(P^{\left(t\right)};H\right)\right\}$ is nondecreasing and bounded above; hence, it converges. Moreover, the overall procedure is an inexact block-coordinate method: $\left\{U_{k}^{\left(t\right)}\right\}$ and $\left\{W_{k}^{\left(t\right)}\right\}$ are updated in closed form, while the transmit update may be inexact due to (i) the diagonal surrogate and (ii) the PAPC dual loop. If the transmit inexactness vanishes asymptotically (e.g., $∥W_{k}^{\left(t\right)}-D_{k}^{\left(t\right)}∥\to 0$, or the hybrid rule selects $P_{\mathrm{WMMSE}}^{\left(t\right)}$ increasingly often), then every accumulation point of $\left\{\left(U^{\left(t\right)},W^{\left(t\right)},P^{\left(t\right)}\right)\right\}$ is a stationary point of the classical WMMSE objective. Otherwise, accumulation points are stationary for the corresponding surrogate objective using $D_{k}=\mathrm{diag}\left(\mathrm{diag}\left(W_{k}\right)\right)$.*

*Sketch. Armijo yields sufficient ascent of WSR(·;H) for accepted feasible iterates, and boundedness follows from PAPCs. Standard inexact-BCD arguments imply convergence of the objective values and stationarity of limit points under vanishing inexactness.*

**Remark** **4** (Practical convergence rate and hybrid impact). *To quantify practical convergence speed and the impact of hybrid switching, we report (i) WSR versus outer iteration and (ii) an iterations-to-target metric (e.g., the number of outer iterations required to reach 99% of the final WSR) under mild and strong spatial correlation. These results complement Proposition 2 by providing an empirical convergence characterization under CSI mismatch.*

**Figure 1:**
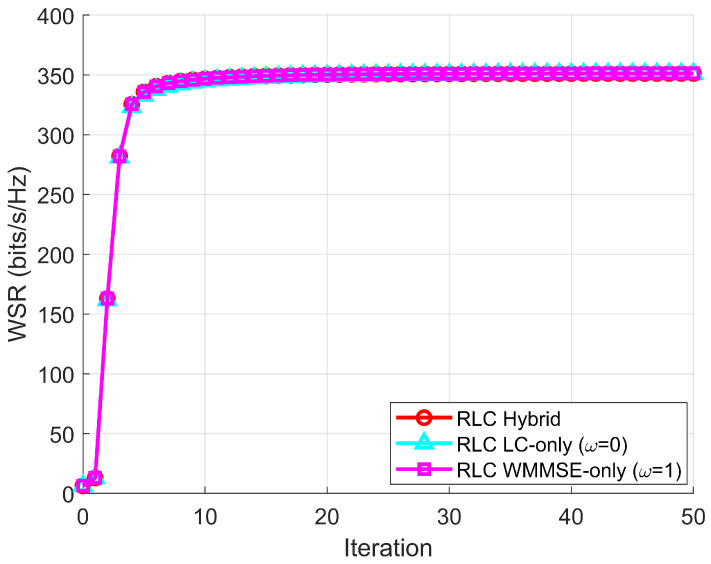
Hybrid-impact ablation under PAPCs and imperfect CSI: WSR versus iteration at SNR=20 dB with correlated channels ($r_{\mathrm{tx}}=0.7$), $\epsilon =10^{-3}$, averaged over 100 trials.

To quantify the impact of the proposed hybrid switching mechanism on convergence under PAPCs and CSI mismatch, we run the same robust algorithm in three modes: hybrid switching, LC-only by fixing $\omega^{\left(t\right)}\equiv 0$, and WMMSE-only by fixing $\omega^{\left(t\right)}\equiv 1$. Figure 1 reports the averaged WSR trajectories versus iteration at SNR=20 dB over spatially correlated channels ($r_{\mathrm{tx}}=0.7$) with CSI error variance $\epsilon =10^{-3}$, averaged over 100 Monte Carlo trials. All three variants converge to essentially the same WSR plateau, indicating that hybrid switching does not compromise the achieved performance in the considered robust setting. The numerical summary is provided in Table 3. The final WSR values are 351.25, 350.95, and 351.29 bps/Hz for hybrid, LC-only, and WMMSE-only, respectively. Moreover, the hybrid mode reaches 99% of its final WSR within 12 iterations, whereas LC-only requires 15 iterations, demonstrating faster practical convergence (relative to the pure LC branch) while preserving stability under imperfect CSI and PAPCs.

**Lemma** **1** (Diagonal-surrogate perturbation bound). *Let $D_{k}=\mathrm{diag}\left(\mathrm{diag}\left(W_{k}\right)\right)$ and $E_{k}=W_{k}-D_{k}$. The diagonal surrogate perturbs the transmit normal matrix by $\Delta A^{\left(t\right)}=\sum_{k=1}^{K}{\hat{H}}_{k}D_{k}^{\left(t\right)}E_{k}^{\left(t\right)}D_{k}^{(t)H}{\hat{H}}_{k}^{H}$, which satisfies $∥\Delta A^{\left(t\right)}{∥}_{2}\;\leq \sum_{k=1}^{K}∥{\hat{H}}_{k}{∥}_{2}^{2}\;∥D_{k}^{\left(t\right)}{∥}_{2}^{2}\;{∥E_{k}^{\left(t\right)}∥}_{2}.$ Hence, the diagonal approximation is accurate when the off-diagonal energy $∥W_{k}^{\left(t\right)}-D_{k}^{\left(t\right)}∥$ is small (diagonal dominance), and it can degrade in strongly coupled regimes (e.g., high SNR / strong correlation), motivating the hybrid safeguard.*

*Implication: Since $∥\Delta A^{\left(t\right)}{∥}_{2}$ scales with $∥W_{k}-{\tilde{W}}_{k}{∥}_{F}$, the diagonal surrogate may be less reliable under strong correlation/high SNR, and the hybrid switching mitigates this by favoring the classical update when needed.*

Diagonal approximation error: To quantify the error introduced by replacing $W_{k}$ with ${\tilde{W}}_{k}≜\mathrm{diag}\left(\mathrm{diag}\left(W_{k}\right)\right)$, we use the relative off-diagonal energy

(39) $$\eta_{k}≜\frac{∥W_{k}-{\tilde{W}}_{k}{∥}_{F}}{∥W_{k}{∥}_{F}},$$

which measures the departure of $W_{k}$ from a diagonal structure. Empirically, $\eta_{k}$ increases under stronger spatial correlation, indicating a less accurate diagonal surrogate.

Moreover, $\eta_{k}$ admits an explicit perturbation bound. Let $E_{k}≜W_{k}-{\tilde{W}}_{k}$ and $\Delta A^{\left(t\right)}≜A^{\left(t\right)}-{\tilde{A}}^{\left(t\right)}=\sum_{k=1}^{K}{\hat{H}}_{k}D_{k}^{\left(t\right)}E_{k}^{\left(t\right)}D_{k}^{(t)H}{\hat{H}}_{k}^{H}$. Then,

(40) $$∥\Delta A^{\left(t\right)}{∥}_{2}\leq \sum_{k=1}^{K}∥{\hat{H}}_{k}D_{k}^{\left(t\right)}{∥}_{2}^{2}\;∥E_{k}^{\left(t\right)}{∥}_{F}=\sum_{k=1}^{K}∥{\hat{H}}_{k}D_{k}^{\left(t\right)}{∥}_{2}^{2}\;\eta_{k}^{\left(t\right)}{∥W_{k}^{\left(t\right)}∥}_{F},$$

so smaller $\eta_{k}$ implies a smaller surrogate perturbation. The hybrid rule further improves robustness by favoring the classical update when the surrogate is less reliable.

PAPC feasibility (every outer iteration): Let $P\left(\lambda \right)={\left(A^{\left(t\right)}+\mathrm{diag}\;\lambda \right)}^{-1}B^{\left(t\right)}$ and update λ by the projected dual step Equations (29) and (30) with the final row-wise safety projection Equation (31). Then, each iterate $P^{\left(t\right)}$ satisfies $\sum_{n}{\left|P_{mn}^{\left(t\right)}\right|}^{2}\leq \rho_{m}$ for all *m* (to numerical tolerance), so PAPCs hold throughout.

### 3.5. Computational Complexity Analysis

In massive MU-MIMO, the computational burden of iterative precoding is often the limiting factor. In particular, the classical WMMSE [23] is dominated per iteration by solving the M×M precoder system in (27), i.e., factorizing $\sigma^{2}I_{M}+\sum_{k=1}^{K}H_{k}U_{k}W_{k}U_{k}^{H}H_{k}^{H}$, which costs $O(M^{3})$. Receiver and weight updates each cost $O(KN^{3})$. Rethinking WMMSE (R-WMMSE) reduces the dominant cost via sketching and matrix-free linear solves and exhibits near-linear scaling in *M* empirically [20].

LC step (Woodbury): Using the stacked channel $\hat{H}={\hat{H}}_{1},\ldots ,{\hat{H}}_{K}]\;\in C^{M\times NK}$ and $S=\mathrm{blkdiag}(S_{1},\ldots ,S_{K})$ with $S_{k}=U_{k}\;\mathrm{diag}\left(W_{k}\right)\;U_{k}^{H}\in C^{N\times N}$, the LC step replaces the M×M inverse by (NK)×(NK) SPD solve via Woodbury:

(41) $$A^{-1}={\left(\sigma^{2}I_{M}+\hat{H}S{\hat{H}}^{H}\right)}^{-1}=\frac{1}{\sigma^{2}}\left[I_{M}-\hat{H}{\left(S^{-1}+\frac{1}{\sigma^{2}}{\hat{H}}^{H}\hat{H}\right)}^{-1}{\hat{H}}^{H}\frac{1}{\sigma^{2}}\right].$$

Algorithm 1 dominant costs per iteration are as follows: (i) Cholesky/solve of $T=S^{-1}+\frac{1}{\sigma^{2}}{\hat{H}}^{H}\hat{H}\in C^{\left(NK)\times (NK\right)}$: $O\;\left({\left(NK\right)}^{3}\right)$; (ii) Gram products with $\hat{H}$ (e.g., ${\hat{H}}^{H}\hat{H}$, ${\hat{H}}^{H}B$): $O\;\left(M{\left(NK\right)}^{2}\right)$; and (iii) per-user N×N factorizations (for $S_{k}^{-1}$): $O(KN^{3})$. Thus, Table 4 shows that the RLC-WMMSE algorithm has dominant cost $O\;\left(M{\left(NK\right)}^{2}\right)+O\;\left({\left(NK\right)}^{3}\right)+O\left(KN^{3}\right)$, which is much smaller than the classical WMMSE algorithm complexity $O(M^{3})$ when M≫NK.
**Algorithm 1** Robust low-complexity WMMSE (RLC-WMMSE) precoding.**Require:** 
Channel estimates ${\left\{{\hat{H}}_{k}\right\}}_{k=1}^{K}$, true channels ${\left\{H_{k}\right\}}_{k=1}^{K}$ (for evaluation), weights ${\left\{\mu_{k}\right\}}_{k=1}^{K}$, noise $\sigma^{2}$, PAPC budgets ρ, max iters *T*, tol ϵ  1:Initialize stacked precoder $P^{\left(0\right)}$ (feasible or normalized)  2:**for** t=1 **to** *T* **do**  3:    **for** k=1 **to***K* **do**  4:        Receive update: $U_{k}^{\left(t\right)}$ by (35),  5:        MSE build: $E_{k}^{\left(t\right)}$ from $U_{k}^{\left(t\right)}$ and $P^{\left(t-1\right)}$  6:        Weight update: $W_{k}^{\left(t\right)}$ by (36),  7:    **end for**  8:    Hybrid switch: compute $\omega^{\left(t\right)}$ by (23),  9:   Classical candidate (with PAPCs): build $A^{\left(t\right)},B^{\left(t\right)}$ by (27); compute $P_{\mathrm{WMMSE}}^{\left(t\right)}\left(\lambda \right)$ via $P\left(\lambda \right)={\left(A^{\left(t\right)}+\mathrm{diag}\lambda \right)}^{-1}B^{\left(t\right)}$ with λ updated by the projected dual step (29) and (30) and the safety projection (31),10:  Low-complexity candidate (Woodbury + PAPCs): form $S^{\left(t\right)}$ and stacked $\hat{H}$ by (25) and (26), compute $P_{\mathrm{LC}}^{\left(t\right)}\left(\lambda \right)$ using the Woodbury closed form by (37) while enforcing PAPCs by (29)–(31) on the LC branch.11:  Hybrid precoder update by (22),12:  Adaptive damping: compute $\alpha^{\left(t\right)}$ by (33), then set $P^{\left(t\right)}=\alpha^{\left(t\right)}P^{\left(t-1\right)}+\left(1-\alpha^{\left(t\right)}\right){\hat{P}}^{\left(t\right)}$13:  Armijo acceptance is evaluated on the PAPC-feasible trial iterate.14:  Evaluate ${\mathrm{WSR}}^{\left(t\right)}$ on true channels $\left\{H_{k}\right\}$ (monotone track); **if** $|{\mathrm{WSR}}^{\left(t\right)}-{\mathrm{WSR}}^{\left(t-1\right)}|<\epsilon$ **then break**15:**end for****Ensure:** 
Per-user blocks ${\left\{P_{k}^{\left(t\right)}\right\}}_{k=1}^{K}$ from the stacked $P^{\left(t\right)}$

#### 3.5.1. PAPC (RLC-WMMSE) Effect

With PAPCs, the classical branch evaluates $P\left(\lambda \right)={\left(A^{\left(t\right)}+\mathrm{diag}\;\lambda \right)}^{-1}B^{\left(t\right)}$ using a short projected dual loop in Equations (29)–(31). In the LC branch, we replace $\sigma^{2}I_{M}$ by the diagonal matrix $A_{\lambda }=\mathrm{diag}\left(\sigma^{2}+\lambda_{m}\right)$ and apply Woodbury with $S^{\left(t\right)}=\mathrm{blkdiag}\left(U_{k}^{\left(t\right)}D_{k}^{\left(t\right)}U_{k}^{(t)H}\right)$ and $\hat{H}=\left[{\hat{H}}_{1},\ldots ,{\hat{H}}_{K}\right]$:

(42) $${\left(A_{\lambda }+\hat{H}S^{\left(t\right)}{\hat{H}}^{H}\right)}^{-1}=A_{\lambda }^{-1}-A_{\lambda }^{-1}\hat{H}\;{\left({\left(S^{\left(t\right)}\right)}^{-1}+{\hat{H}}^{H}A_{\lambda }^{-1}\hat{H}\right)}^{\;-1}\;{\hat{H}}^{H}A_{\lambda }^{-1}.$$

The per-iteration asymptotic costs remain $O\;\left(M{\left(NK\right)}^{2}\right)+O\;\left({\left(NK\right)}^{3}\right)+O\left(KN^{3}\right)$; the dual loop adds *J* cheap inner steps (typically J≤5), each requiring one (M×NK) triangular solve and power check, i.e., an extra $O\;\left(J\;M(NK)\right)$ that is lower-order when M≫NK.

*Takeaway.* Because NK≪M in massive MU-MIMO, LC/RLC replace the cubic $O(M^{3})$ term with operations that scale with NK. Empirically, we observe a crossover where LC/RLC become faster than classical WMMSE when M≳c(NK) (with c≈3 in our setup), and the PAPC dual loop incurs only a small constant overhead. Imperfect CSI (updates on $\hat{H}$) does not change orders of complexity.

#### 3.5.2. Practical Implementation and Latency Considerations

We emphasize that RLC-WMMSE remains an iterative optimizer and thus is not primarily intended to replace one-shot closed-form precoders in extremely stringent URLLC pipelines. Rather, it targets regimes in which a small iteration budget is feasible within the channel coherence time and the dominant computations can be efficiently parallelized. In practice, the latency can be reduced by warm-starting from the previous slot/TTI solution, adopting early stopping with a fixed iteration cap or an iterations-to-target criterion (e.g., $t_{99}$), and implementing the dominant (NK)×(NK) SPD solves using highly optimized batched Cholesky/LDL routines on GPU/FPGA/ASIC. From this perspective, the key advantage of the proposed reformulation is that it shifts the dominant per-iteration cost from an M×M inversion to an (NK)×(NK) SPD solve, which is more favorable when M≫NK.

### 3.6. Implementation Considerations and Signaling Overhead Analysis

At iteration *t*, the BS needs downlink (DL) CSI and the current receive filters and weights for each user. In a TDD deployment, the BS estimates the downlink channels $\left\{{\hat{H}}_{k}\right\}$ from uplink pilots by reciprocity and can update $U_{k}^{\left(t\right)}$ and $W_{k}^{\left(t\right)}$ locally. Hence, no per-iteration DL feedback is required beyond the usual pilot overhead, and the additional PAPC dual variables λ are kept entirely at the BS. In an FDD system, the user equipments (UEs) estimate the channels from DL pilots and must feed back effective CSI based on ${\hat{H}}_{k}$ for the RLC-WMMSE updates. A full WMMSE mode would return the receive filters $U_{k}^{\left(t\right)}\;\in \;C^{N\times d_{k}}$ and the Hermitian weight matrices $W_{k}^{\left(t\right)}\;\in \;C^{d_{k}\times d_{k}}$. Using $b_{c}$ bits per complex number and $b_{r}$ bits per real number, the per-user payload per iteration is on the order of

(43) $$B_{\mathrm{full},k}\approx Nd_{k}b_{c}+\frac{d_{k}\left(d_{k}+1\right)}{2}b_{r},$$

where $\frac{d_{k}\left(d_{k}+1\right)}{2}$ real entries describe a $d_{k}\times d_{k}$ Hermitian matrix. Our LC branch suggests a lighter diagonal feedback mode. Since the algorithm only uses the diagonal surrogate $D_{k}^{\left(t\right)}=\mathrm{diag}\left(\mathrm{diag}\left(W_{k}^{\left(t\right)}\right)\right)$, the UE can quantize and feed back $\mathrm{diag}\left(W_{k}^{\left(t\right)}\right)\;\in \;R^{d_{k}}$ together with a compressed representation of $U_{k}^{\left(t\right)}$ (e.g., a codebook index or a few dominant singular vectors). The overhead reduces to roughly

(44) $$B_{\mathrm{LC},k}\approx d_{k}b_{r}+B_{\mathrm{codebook}},$$

which can be significantly smaller than $B_{\mathrm{full},k}$ when $d_{k}\ll N$. In both modes, the additional PAPC dual variables are updated at the BS and incur no extra DL/UL signaling.

*Relation to imperfect CSI model: In our stochastic CSI model $H_{k}={\hat{H}}_{k}+\Delta_{k}$, the mismatch variance ε captures the combined effect of channel estimation noise and, in FDD, feedback quantization/compression and delay. Longer UL/DL pilot sequences or finer feedback quantization reduce ε, while aggressive compression in the LC feedback mode typically increases it. The proposed RLC-WMMSE algorithm is designed to be robust to such mismatches, as reflected in the simulations of Section 4.*

## 4. Simulations and Results

### 4.1. Simulation Setup

We examine a single-cell massive MU-MIMO downlink where a BS with *M* transmit antennas serves *K* users. Each user is equipped with *N* receive antennas and is allocated $d_{k}=N$ data streams. Under the sum-power constraint (SPC), the BS transmit-power budget is set to $P_{\max}=10\;W$. For per-antenna power constraints, the power limits are collected in $\rho ={\left[\rho_{1},\ldots ,\rho_{M}\right]}^{T}$ with $\rho_{m}=P_{\max}/M$. The channel matrices $\left\{H_{k}\right\}$ are generated as circularly symmetric complex Gaussian fading with large-scale pathloss $128.1+37.6\log_{10}\left(d\right)\;\mathrm{dB}$ [30], where d∈[0.1,0.3]km denotes the user–BS distance. The noise variance is identical for all users and is set to $\sigma^{2}=\frac{P_{\max}}{10^{\mathrm{SNR}/10}}$, so that SNR (in dB) corresponds to the average received SNR in the absence of precoding.

Unless stated otherwise, results are reported under perfect CSI at the BS and are averaged over 100 independent channel realizations. For robustness evaluations, we explicitly model imperfect CSI via ${\hat{H}}_{k}=H_{k}+\Delta_{k}$, where $\Delta_{k}∼\mathrm{CN}\left(0,\varepsilon I\right)$ and ε controls the estimation NMSE. Throughout the simulations, we use $\kappa =10^{-3}$, $\eta =10^{-3}$, $\alpha_{\min}=0.2$, $\alpha_{\max}=0.9$, tolerance $\epsilon =10^{-4}$, and a maximum of T=100 iterations. All experiments are implemented in Matlab R2024b on a Windows 11 (64-bit) workstation with an Intel i7-12700H CPU at 3.20 GHz, 16 GB RAM, and RTX Graphics.

### 4.2. Robust Low-Complexity (RLC-WMMSE) Performance Under Correlated Channels

This subsection presents simulation results that evaluate the performance of the proposed RLC-WMMSE algorithm under per-antenna power constraints and correlated-channel conditions. The method is compared with several baseline schemes: the LC-WMMSE algorithm [25], the classical WMMSE algorithm [23], the R-WMMSE approach [20], and two non-iterative precoding techniques—zero-forcing (ZF) [31] and block diagonalization (BD) [32]. The closed-form ZF and BD methods exploit low-dimensional channel properties (e.g., BD uses null-space projection to suppress interference) and exhibit low computational complexity, specifically $O(K^{3})$ for ZF and $O(KM^{2})$ for BD [31,32]. Although these non-iterative schemes are computationally efficient and practical for massive MU-MIMO, they generally yield suboptimal weighted sum-rate performance. To ensure a fair comparison, each trial initializes the precoder as $P^{\left(0\right)}∼\mathrm{CN}\left(0,1\right)$, which is then scaled to satisfy the power constraints and kept identical for all methods. To investigate robustness in realistic propagation environments, we first consider on spatially correlated channels at the BS. For user *k*, the true downlink channel is generated according to

(45) $${\hat{H}}_{k}=R_{\mathrm{BS}}^{1/2}W_{k},\;W_{k}∼\mathrm{CN}\left(0,I_{M\times N}\right),$$

where $R_{\mathrm{BS}}\in C^{M\times M}$ is the BS correlation matrix. We adopt an exponential model ${\left[R_{\mathrm{BS}}\right]}_{m,n}=r^{\left|m-n\right|}$ with 0≤r<1 and use r=0.7 in the correlated-channel experiments. The i.i.d. Rayleigh case is recovered by setting $R_{\mathrm{BS}}=I_{M}$. Apart from the choice of $R_{\mathrm{BS}}$, the simulation protocol (SNR grid, (M,N,K), initialization, tolerances, and power normalization) is identical for both correlated and i.i.d. channels.

**Figure 2:**
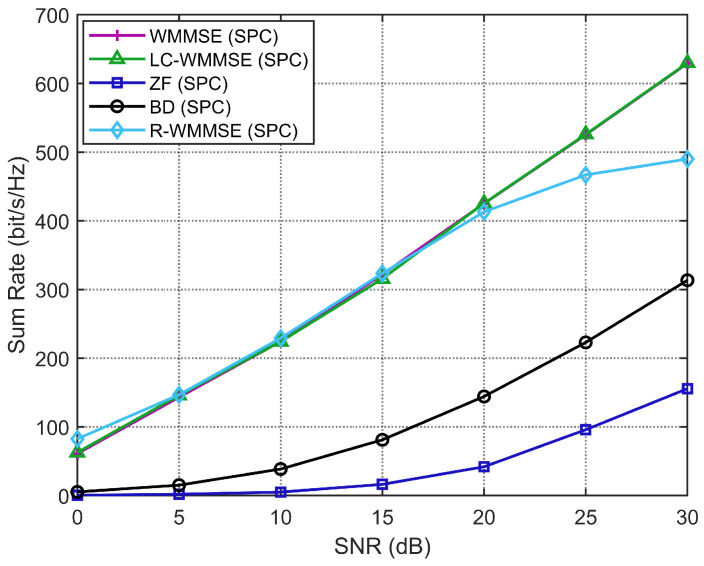
WSR of MU-MIMO systems with perfect CSI (M=128, K=16, N=4).

Figure 2 shows the WSR versus SNR for correlated MU-MIMO channels with perfect CSI. The LC-WMMSE algorithm closely matches the WSR of the classical WMMSE algorithm over the whole SNR range, while both clearly outperform the R-WMMSE and the closed-form ZF and BD precoders.

**Figure 3:**
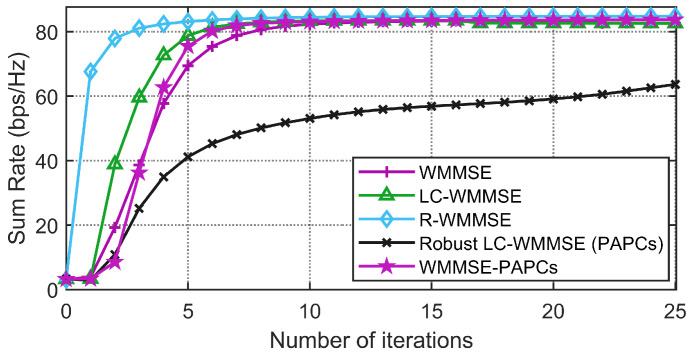
Convergence comparison between the proposed RLC-WMMSE algorithm and the WMMSE algorithms with PAPCs under M=64, K=12, N=2, and SNR=10dB.

**Figure 4:**
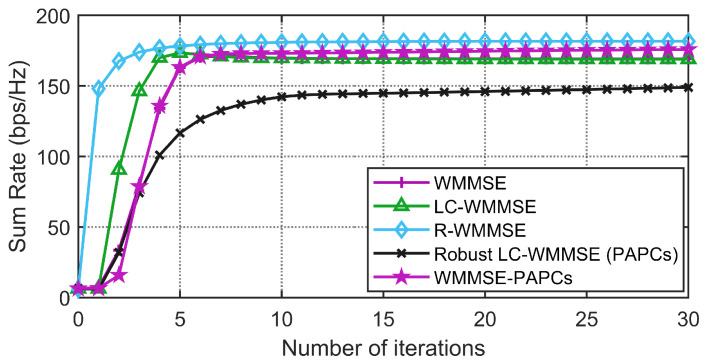
Convergence comparison between the proposed RLC-WMMSE algorithm and the WMMSE algorithms with PAPCs under M=128, K=16, N=4, 0dB), and SNR=0dB.

Figure 3 and Figure 4 illustrate the convergence behavior of the different PAPC schemes for two system dimensions, (M,K,N)=(64,12,2) and (128,16,4), at SNR=10dB,0dB. For the SPC case, WMMSE, LC-WMMSE, and R-WMMSE all exhibit fast, monotone convergence and attain almost identical final WSR, with R-WMMSE and LC-WMMSE reaching the steady state in noticeably fewer iterations than classical WMMSE. Under PAPCs, the proposed robust LC-WMMSE converges much faster than the WMMSE-PAPCs baseline while achieving nearly the same steady-state WSR, confirming that the low-complexity Woodbury/dual design preserves good convergence properties even in the presence of per-antenna constraints.

**Figure 5:**
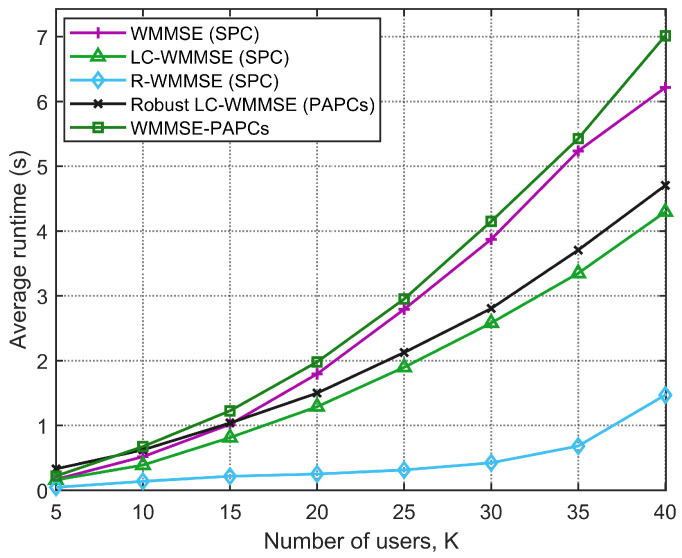
Average CPU convergence time versus number of users *K* (M=128, N=2, SNR=10 dB).

Now, we compare our proposed RLC-WMMSE algorithm with the LC-WMMSE algorithm, the classical WMMSE and R-WMMSE schemes in terms of average CPU time to convergence, and quantify the additional overhead introduced by the PAPC variants (robust LC-WMMSE and WMMSE-PAPCs). Figure 5 shows the runtime versus the number of users *K* for a system with M=128 BS antennas, N=2 receive antennas per user, and SNR=10 dB. As *K* increases, all algorithms exhibit higher computational cost, but with markedly different slopes. The proposed RLC-WMMSE algorithm is consistently faster than the classical WMMSE baseline, showing a much flatter growth in runtime as *K* grows, which is consistent with its reduced per-iteration complexity. For the largest tested value K=40, LC-WMMSE is roughly 40% faster than WMMSE in terms of CPU time, whereas R-WMMSE, thanks to its randomized/sketched updates, achieves runtimes that are almost an order of magnitude smaller than those of WMMSE. The robust LC-WMMSE under PAPCs remains significantly cheaper than the exact WMMSE-PAPCs solver, confirming the benefit of the proposed dual-based low-complexity design for PAPCs.

**Figure 6:**
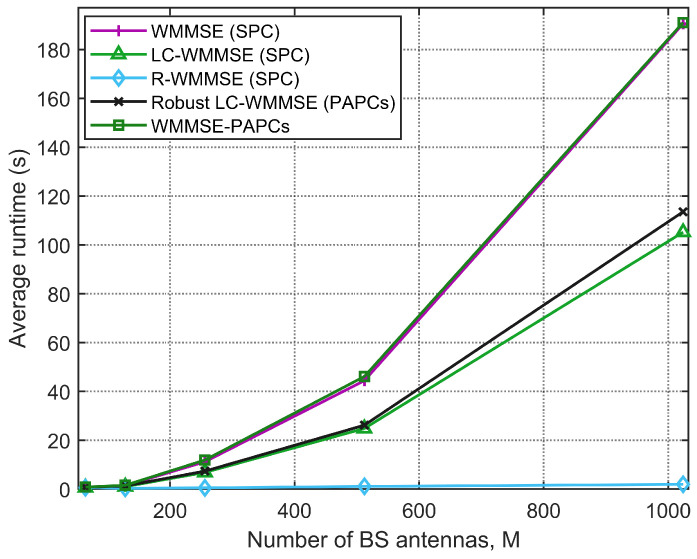
Average CPU convergence time versus number of BS antennas *M* (K=16, N=4, 10dB).

Figure 6 shows the runtime as a function of the number of BS antennas *M* for K=16 users, N=4 receive antennas per user, and SNR=10dB. The computational cost of all methods increases with *M*, but the scaling behavior differs. The classical WMMSE and WMMSE-PAPCs curves grow rapidly, reflecting the $O(M^{3})$ matrix inversions in each iteration. In contrast, LC-WMMSE and robust LC-WMMSE grow much more slowly with *M*, and for $M\approx 10^{3}$, the LC-WMMSE achieves about a 1.7× speedup over WMMSE while remaining substantially faster than WMMSE-PAPCs. R-WMMSE exhibits the lowest runtime across all antenna dimensions, in line with its near-linear complexity in *M*. Overall, these results confirm the complexity analysis: LC-WMMSE provides a substantial reduction in computational cost compared with the classical WMMSE algorithm, and the robust LC-WMMSE inherits this favorable scaling even in the presence of PAPCs.

**Figure 7:**
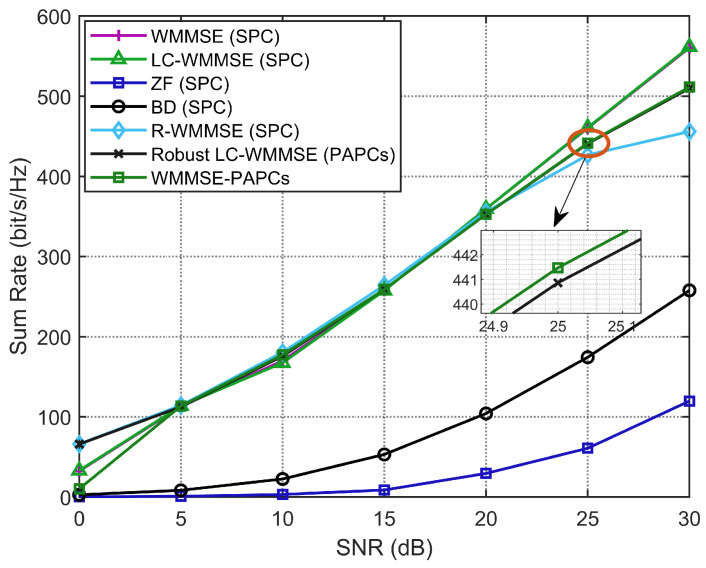
WSR performance with different SNRs (M=128, K=16, N=4).

Figure 7 compares the weighted sum-rate versus SNR for the considered precoders with correlated channels (M=128, K=16, N=4, and imperfect CSI). As expected, all schemes benefit from increasing SNR, but their relative gaps remain almost constant. The benchmark WMMSE-PAPCs and its normalized variant achieve the highest WSR, and the proposed RLC-WMMSE closely tracks both curves across the entire SNR range. At SNR=30 dB, for example, the WSR loss of RLC-WMMSE with respect to WMMSE-PAPCs is below 0.3%, and below 0.2% relative to the normalized WMMSE-PAPCs baseline. R-WMMSE delivers slightly lower WSR due to the sketching approximation, whereas LC-WMMSE (SPC), ZF, and BD exhibit larger gaps, highlighting the importance of both PAPC enforcement and robust design under CSI mismatch.

**Figure 8:**
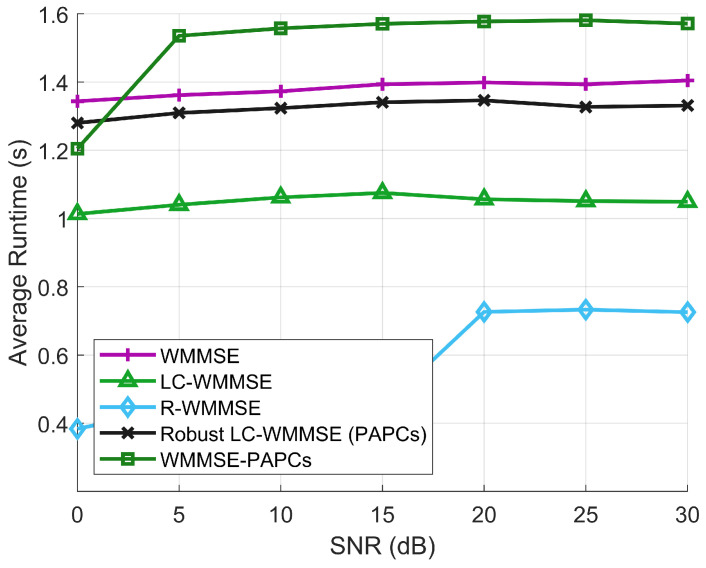
Average runtime between different SNRs.

Figure 8 reports the corresponding average runtimes per channel realization versus SNR. The runtimes of all algorithms are essentially insensitive to SNR and are dominated by their per-iteration matrix operations. R-WMMSE is the fastest method, but this comes at the cost of a noticeable WSR loss. LC-WMMSE reduces the runtime by about 20–25% compared to classical WMMSE, confirming the effectiveness of the Woodbury reformulation. The proposed RLC-WMMSE incurs only a moderate overhead relative to LC-WMMSE to enforce PAPCs and handle imperfect CSI, yet it still runs about 10–15% faster than the full WMMSE-PAPCs solver. Overall, these results demonstrate that RLC-WMMSE attains WSR performance essentially identical to the best PAPC baselines while offering more favorable runtime scaling than classical WMMSE and WMMSE-PAPCs in the large-array regime.

### 4.3. Robustness to Channel Estimation Errors

In this subsection we investigate the robustness of the proposed robust LC-WMMSE precoder to imperfect CSI at the BS. For each user *k*, the estimated downlink channel is modeled as ${\hat{H}}_{k}=H_{k}+E_{k}$, where $H_{k}\in C^{M\times N}$ is the true channel matrix and $E_{k}$ collects the estimation errors whose entries are i.i.d. circularly symmetric complex Gaussian. The quality of the CSI is parameterized by the normalized mean-squared error (NMSE)

(46) $$\epsilon ≜\frac{E\;\left[{∥{\hat{H}}_{k}-H_{k}∥}_{F}^{2}\right]}{E\;\left[{∥H_{k}∥}_{F}^{2}\right]},$$

so that ϵ=0 corresponds to perfect CSI and larger values of ϵ represent increasingly severe estimation errors. For each fixed ϵ, we generate $\left(H_{k},{\hat{H}}_{k}\right)$ according to the above model and compare the following transceiver designs:

- WMMSE (oracle, H): The classical WMMSE algorithm with a sum-power constraint, which has perfect knowledge of $H_{k}$ and serves as an upper bound.
- WMMSE (mismatch, $\hat{H}\;\to \;H$): The classical WMMSE algorithm that designs the precoder using the imperfect CSI ${\hat{H}}_{k}$, while the achieved weighted sum-rate is always evaluated on the true channels $H_{k}$.
- Robust LC-WMMSE (PAPCs): The proposed low-complexity LC-WMMSE algorithm with per-antenna power constraints, which updates all variables using only ${\hat{H}}_{k}$ and is also evaluated on the true channels $H_{k}$.

Let $R_{\mathrm{or}}\left(\epsilon \right)$, $R_{\mathrm{mis}}\left(\epsilon \right)$, and $R_{\mathrm{RLC}}\left(\epsilon \right)$ denote the average weighted sum-rate (WSR) of the oracle WMMSE, mismatched WMMSE, and robust LC-WMMSE, respectively, for a given value of ϵ. The relative WSR loss with respect to the oracle design is defined as

(47) $$\Delta_{\mathrm{mis}}\left(\epsilon \right)=\frac{R_{\mathrm{or}}\left(\epsilon \right)-R_{\mathrm{mis}}\left(\epsilon \right)}{R_{\mathrm{or}}\left(\epsilon \right)}\times 100\%,\;\Delta_{\mathrm{RLC}}\left(\epsilon \right)=\frac{R_{\mathrm{or}}\left(\epsilon \right)-R_{\mathrm{RLC}}\left(\epsilon \right)}{R_{\mathrm{or}}\left(\epsilon \right)}\times 100\%.$$

**Figure 9:**
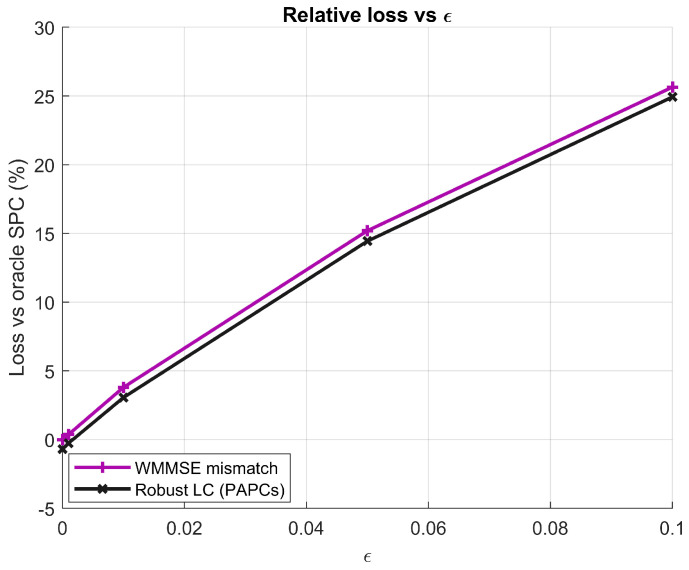
WSR versus channel estimation NMSE parameter ϵ at M=128, K=16, N=4, SNR=10 dB. The oracle WMMSE (perfect CSI), mismatched WMMSE (imperfect CSI), and robust LC−WMMSE with PAPCs are compared.

**Figure 10:**
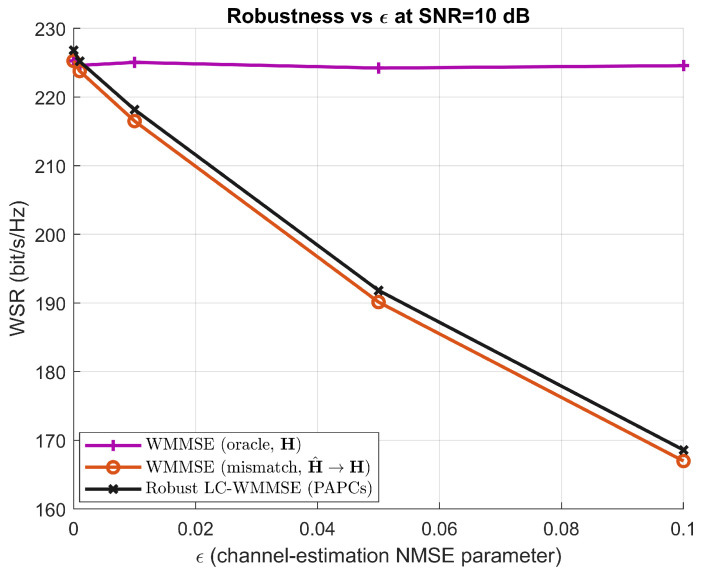
Relative WSR loss with respect to the oracle WMMSE baseline versus channel estimation NMSE parameter ϵ at M=128, K=16, N=4, SNR=10 dB.

Figure 9 shows the WSR versus ϵ at SNR=10 dB. As expected, the oracle WMMSE curve is almost insensitive to ϵ, since it always uses the true CSI. In contrast, the mismatched WMMSE suffers a significant performance degradation as ϵ increases. The proposed robust LC-WMMSE (PAPCs) consistently tracks the oracle curve more closely, thereby mitigating the loss induced by imperfect CSI. The relative losses $\Delta_{\mathrm{mis}}\left(\epsilon \right)$ and $\Delta_{\mathrm{RLC}}\left(\epsilon \right)$ are shown in Figure 10; these results confirm that the proposed design reduces the WSR loss compared to the mismatched WMMSE baseline over the entire range of channel-estimation errors considered.

**Figure 11:**
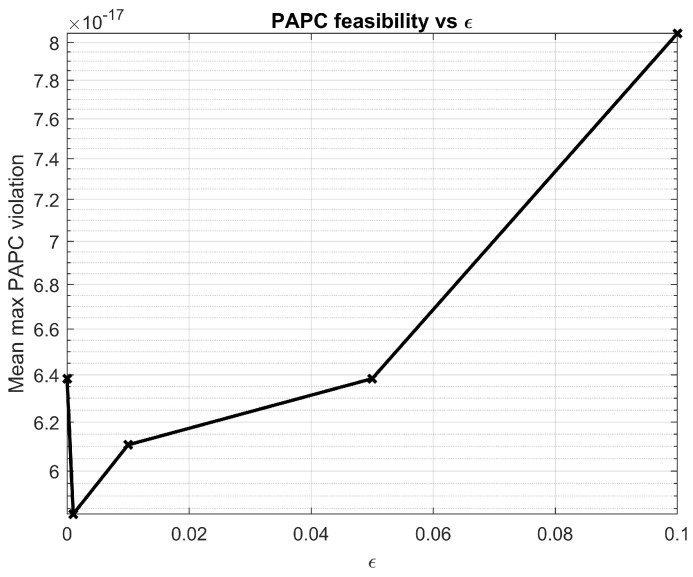
Average maximum PAPC violation versus channel estimation NMSE parameter ϵ for the proposed robust LC−WMMSE precoder at M=128, K=16, N=4, SNR=10 dB.

To verify that robustness does not come at the expense of violating the per-antenna power constraints, we also monitor the average maximum PAPC violation

(48) $$\bar{v}\left(\epsilon \right)≜E\;\left[\max_{1\leq m\leq M}{\left(∥p_{m}{∥}_{2}^{2}-\rho_{m}\right)}_{+}\right],$$

where $p_{m}$ denotes the *m*th row of the final precoder and ${\left(x\right)}_{+}≜\max\left\{x,0\right\}$. As illustrated in Figure 11, the proposed robust LC-WMMSE scheme satisfies the PAPCs up to numerical precision for all tested values of ϵ.

### 4.4. Performance Under i.i.d. Rayleigh

To provide a baseline reference, we first consider an uncorrelated i.i.d. Rayleigh fading scenario with imperfect CSI at the BS. The true downlink channels are drawn as $H_{k}∼\mathrm{CN}\left(0,I_{M}\right)$, while the BS only has access to the estimates ${\hat{H}}_{k}=H_{k}+E_{k}$, where $E_{k}∼\mathrm{CN}\left(0,\epsilon \;I_{M}\right)$ models the estimation error. In this subsection, we fix the NMSE parameter to $\epsilon =10^{-3}$ (approximately −30 dB channel-estimation NMSE), and set the transmit-side correlation coefficient to $r_{\mathrm{tx}}=0$. Unless otherwise stated, we use M=128 BS antennas and K=16 users, each with N=4 receive antennas (and streams), and a total BS sum power ρ=10 W under the SPC case. For PAPC-based schemes, the per-antenna budgets are chosen as $\rho_{m}=\rho /M$ so that $\sum_{m=1}^{M}\rho_{m}=\rho$. All results are averaged over a large number of independent Monte Carlo channel realizations.

**Figure 12:**
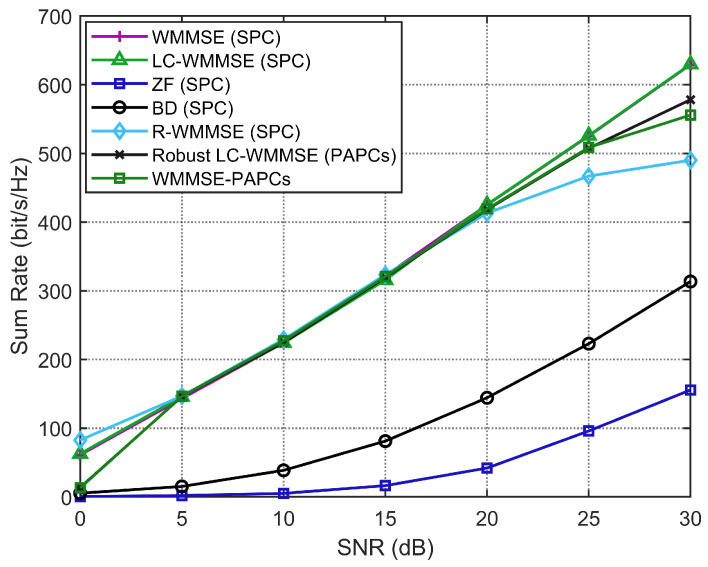
Sum-rate versus SNR for the i.i.d. Rayleigh scenario (M=128, K=16, N=4, $\epsilon =10^{-3}$, $r_{\mathrm{tx}}=0$).

Figure 12 shows the weighted sum-rate versus SNR for the i.i.d. Rayleigh case with imperfect CSI ($\epsilon =10^{-3}$, $r_{\mathrm{tx}}=0$). The proposed LC-WMMSE and robust LC-WMMSE (PAPCs) closely track the classical WMMSE and WMMSE-PAPCs benchmarks, respectively, across the entire SNR range.

**Figure 13:**
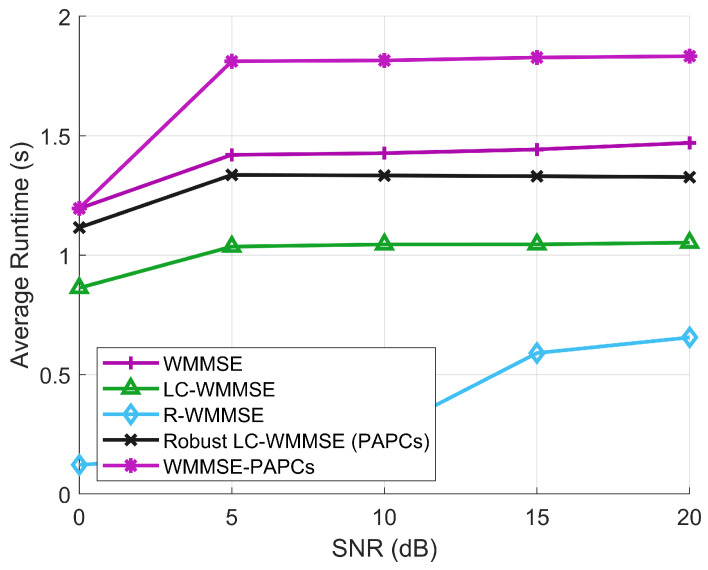
Average runtime between different SNRs under the i.i.d. Rayleigh scenario (M=128, K=16, N=4).

Figure 13 reports the corresponding average runtime per channel realization, highlighting the significant complexity reduction of LC-WMMSE and robust LC-WMMSE compared with the WMMSE algorithm and WMMSE-PAPCs algorithm counterparts.

## 5. Conclusions

Weighted sum-rate maximization in downlink massive MU-MIMO with per-antenna power constraints (PAPCs) and imperfect CSI is computationally demanding, particularly for classical WMMSE algorithms whose cost scales cubically with the number of base-station antennas. This paper proposed a robust low-complexity WMMSE (RLC-WMMSE) precoding framework that preserves the favorable WSR performance of classical WMMSE while explicitly enforcing PAPCs under stochastic CSI mismatch. The approach combines a Woodbury-based low-complexity transmit update that operates on an (NK)×(NK) SPD system, a hybrid switching rule with adaptive damping that blends classical and low-complexity updates, and a lightweight diagonal dual regularization with row-wise safety projection to satisfy per-antenna power limits.

A qualitative complexity analysis shows that the proposed RLC-WMMSE scheme becomes more efficient than classical WMMSE once the number of BS antennas significantly exceeds the total number of data streams, which is typical in massive MU-MIMO deployments. Extensive simulations over i.i.d. and correlated channels, and for various CSI mismatch levels, demonstrate that RLC-WMMSE attains WSR performance very close to a WMMSE-PAPCs benchmark, while maintaining negligible average PAPC violations and offering clearly favorable runtime scaling as the array size grows. The implementation and signaling discussion further indicates that robustness and PAPC feasibility can be handled entirely at the BS, with only mild feedback requirements on the user side.

Overall, the proposed RLC-WMMSE method provides a practical, feasibility-aware, and computationally efficient precoding option for large-array base stations with per-antenna power budgets and imperfect CSI. Future work will consider extensions to multi-cell coordination, hybrid analog–digital architectures, and more refined stochastic error models that capture channel aging and hardware impairments.

## Figures and Tables

**Table 1:** Comparison of WMMSE variants. SPD: symmetric positive definite.

	R-WMMSE	LC-WMMSE	RLC-WMMSE (Proposed)
Principle	Randomized sketching	Structure exploitation (Woodbury + $\mathrm{diag}(W_{k})$)	Robust structure exploitation (Woodbury + $\mathrm{diag}(W_{k})$ + dual PAPCs)
Update size	Compressed (sketched domain)	(NK)×(NK) SPD solve	(NK)×(NK) SPD solve with dual variables
CSI/constraints	Perfect CSI, SPC	Perfect CSI, SPC	Imperfect CSI (stochastic mismatch), PAPCs
Dominant cost	Sketch products + small SPD solve	Build A,B + (NK)×(NK) SPD solve	LC cost + short dual loop (*J* inner steps)
Error source	Sketching bias/variance	Neglect of off-diagonals in $W_{k}$	Diagonal surrogate + inexact dual PAPC enforcement

**Table 2:** Summary of notation. Bold denotes matrices/vectors; ${\left(\cdot \right)}^{H}$ is the Hermitian transpose and ${∥\cdot ∥}_{F}$ the Frobenius norm.

Notation	Meaning
*M*	Number of BS transmit antennas
*N*	Number of receive antennas per user
*K*	Number of users
$d_{k}$	Number of streams for user *k*
$D≜\sum_{k=1}^{K}d_{k}$	Total number of streams
$H_{k}\in C^{M\times N}$	True downlink channel from BS to user *k*
${\hat{H}}_{k}\in C^{M\times N}$	Estimated channel for user *k* (imperfect CSI)
$\Delta_{k}$	Channel estimation error for user *k* with $H_{k}={\hat{H}}_{k}+\Delta_{k}$
ε	Estimation NMSE parameter in the stochastic CSI model
$P_{k}\in C^{M\times d_{k}}$	Precoder for user *k*
$P=\left[P_{1},\ldots ,P_{K}\right]\in C^{M\times D}$	Stacked precoder (all users)
$s_{k}\in C^{d_{k}}$	Data symbol vector for user *k*
$x\in C^{M}$	Transmit signal at the BS
$y_{k}\in C^{N}$	Received signal at user *k*
$n_{k}\in C^{N}$	AWGN at user *k*
$\sigma^{2}$	Noise power (per receive antenna)
$P_{\max}$	Total transmit power budget under SPC
$\rho ={\left[\rho_{1},\ldots ,\rho_{M}\right]}^{T}$	Per-antenna power budgets (PAPCs)
$P_{\mathrm{PAPC}}$	Feasible precoder set satisfying the per-antenna power constraints
$U_{k}\in C^{N\times d_{k}}$	MMSE receive filter for user *k*
$W_{k}\in C^{d_{k}\times d_{k}}$	Weight matrix for user *k*
$E_{k}\in C^{d_{k}\times d_{k}}$	MSE matrix for user *k*
$D_{k}=\mathrm{diag}\left(\mathrm{diag}\left(W_{k}\right)\right)$	Diagonal weight surrogate used in LC/RLC branch
$S_{x}=\sum_{j=1}^{K}P_{j}P_{j}^{H}\in C^{M\times M}$	BS transmit covariance matrix
$H=\left[H_{1},\ldots ,H_{K}\right]\in C^{M\times (NK)}$	Stacked true channel
$\hat{H}=\left[{\hat{H}}_{1},\ldots ,{\hat{H}}_{K}\right]\in C^{M\times (NK)}$	Stacked estimated channel
$S=\mathrm{blkdiag}\left(U_{1}D_{1}U_{1}^{H},\ldots ,U_{K}D_{K}U_{K}^{H}\right)\in C^{NK\times NK}$	Block-diagonal weight matrix (LC/RLC branch)
$B_{\mathrm{LC}}=\left[\;H_{1}U_{1}D_{1},\ldots ,H_{K}U_{K}D_{K}\;\right]\in C^{M\times D}$	RHS factor for LC/RLC Woodbury update (true channel)
$B_{\mathrm{class}}=\left[\;H_{1}U_{1}W_{1},\ldots ,H_{K}U_{K}W_{K}\;\right]\in C^{M\times D}$	RHS factor for classical WMMSE update
$G=H^{H}H\in C^{\left(NK)\times (NK\right)}$	Stacked Gram matrix
$A^{\left(t\right)},\;B^{\left(t\right)}$	Quadratic WMMSE transmit matrices at iteration *t*
$\lambda^{\left(t\right)}\in R_{+}^{M}$	Dual variables for PAPCs at iteration *t*
$A_{\lambda }^{\left(t\right)}=\sigma^{2}I_{M}+\mathrm{diag}\left(\lambda^{\left(t\right)}\right)$	PAPC-regularized diagonal matrix in the classical branch
$I_{M},\;I_{N},\;I_{d_{k}}$	Identity matrices of sizes *M*, *N*, and $d_{k}$
diag(·),blkdiag(·),tr(·)	Standard matrix operators (diagonal, block-diagonal, trace)
$P_{\mathrm{WMMSE}}^{\left(t\right)}$	Classical WMMSE precoder at iteration *t*
$P_{\mathrm{LC}}^{\left(t\right)}$	LC-WMMSE (Woodbury) precoder at iteration *t*
$P_{\mathrm{RLC}}^{\left(t\right)}$	Robust LC-WMMSE (with PAPCs and imperfect CSI) precoder at iteration *t*
$\omega^{\left(t\right)}$	Hybrid switching factor at iteration *t*
$\alpha^{\left(t\right)}$	Adaptive damping factor at iteration *t*
${\mathrm{WSR}}^{\left(t\right)}$	Weighted sum-rate at iteration *t* (bps/Hz)
*J*	Number of dual inner steps

**Table 3:** Hybrid-impact ablation summary at SNR =20 dB, $r_{tx}=0.7$, $\epsilon =10^{-3}$ (100 trials).

Variant	Final WSR (bps/Hz)	$t_{99}$ (Iterations)
RLC Hybrid	351.25	12
RLC LC-only (ω=0)	350.95	15
RLC WMMSE-only (ω=1)	351.29	12

**Table 4:** Per-iteration computational complexity.

Operation	Classical WMMSE	LC-WMMSE (SPC)	RLC-WMMSE (PAPC)
Precoder solve	$O(M^{3})$	$O\;\left(M{\left(NK\right)}^{2}\right)+O\;\left({\left(NK\right)}^{3}\right)$	same as LC + dual $\;+\;O\;\left(J\;M(NK)\right)$
Per-user N×N factorizations	$O(KN^{3})$	$O(KN^{3})$	$O(KN^{3})$
Gram products ($H^{H}H$, $H^{H}B$)	$O\;\left(M{\left(NK\right)}^{2}\right)$	$O\;\left(M{\left(NK\right)}^{2}\right)$	$O\;\left(M{\left(NK\right)}^{2}\right)$
Hybrid switch $\omega^{\left(t\right)}$	–	$O(KN^{2})$	$O(KN^{2})$
Adaptive damping $\alpha^{\left(t\right)}$	–	O(1)	O(1)
Total (dominant)	$O(M^{3}+KN^{3})$	$O\;\left(M{\left(NK\right)}^{2}+{\left(NK\right)}^{3}+KN^{3}\right)$	$O\;\left(M{\left(NK\right)}^{2}+{\left(NK\right)}^{3}+KN^{3}\right)$

## Data Availability

The original contributions presented in this study are included in the article. Further inquiries can be directed to the corresponding author.
